# The Impact of Insertion Sequences on O-Serotype Phenotype and Its O-Locus-Based Prediction in *Klebsiella pneumoniae* O2 and O1

**DOI:** 10.3390/ijms21186572

**Published:** 2020-09-08

**Authors:** Daria Artyszuk, Radosław Izdebski, Anna Maciejewska, Marta Kaszowska, Aleksandra Herud, Valeria Szijártó, Marek Gniadkowski, Jolanta Lukasiewicz

**Affiliations:** 1 Ludwik Hirszfeld Institute of Immunology and Experimental Therapy, Polish Academy of Sciences, Laboratory of Microbial Immunochemistry and Vaccines, 53-114 Wroclaw, Poland; daria.artyszuk@hirszfeld.pl (D.A.); anna.maciejewska@hirszfeld.pl (A.M.); marta.kaszowska@hirszfeld.pl (M.K.); 2 Department of Molecular Microbiology, National Medicines Institute, 00-725 Warsaw, Poland; r.izdebski@nil.gov.pl (R.I.); m.gniadkowski@nil.gov.pl (M.G.); 3 Ludwik Hirszfeld Institute of Immunology and Experimental Therapy, Polish Academy of Sciences, Laboratory of Genomics & Bioinformatics, 53-114 Wroclaw, Poland; aleksandra.herud@hirszfeld.pl; 4 Arsanis Biosciences GmbH, 1030 Vienna, Austria; valeria.szijarto@cebina.eu

**Keywords:** *Klebsiella*, O-antigen, lipopolysaccharide, LPS, O-serotype, NMR, WGS, kaptive

## Abstract

*Klebsiella pneumoniae* is a nosocomial pathogen, pointed out by the World Helth Organisation (WHO) as “critical” regarding the highly limited options of treatment. Lipopolysaccharide (LPS, O-antigen) and capsular polysaccharide (K-antigen) are its virulence factors and surface antigens, determining O- and K-serotypes and encoded by O- or K-loci. They are promising targets for antibody-based therapies (vaccines and passive immunization) as an alternative to antibiotics. To make such immunotherapy effective, knowledge about O/K-antigen structures, drift, and distribution among clinical isolates is needed. At present, the structural analysis of O-antigens is efficiently supported by bioinformatics. O- and K-loci-based genotyping by polymerase chain reaction (PCR) or whole genome sequencing WGS has been proposed as a diagnostic tool, including the Kaptive tool available in the public domain. We analyzed discrepancies for O2 serotyping between Kaptive-based predictions (O2 variant 2 serotype) and the actual phenotype (O2 variant 1) for two *K. pneumoniae* clinical isolates. Identified length discrepancies from the reference O-locus resulted from insertion sequences (ISs) within *rfb* regions of the O-loci. In silico analysis of 8130 O1 and O2 genomes available in public databases indicated a broader distribution of ISs in *rfb*s that may influence the actual O-antigen structure. Our results show that current high-throughput genotyping algorithms need to be further refined to consider the effects of ISs on the LPS O-serotype.

## 1. Introduction

*Klebsiella pneumoniae* is a Gram-negative bacterium which is part of the human microbiota; however, it is also a frequent cause of nosocomial and community-acquired infections in newborns, the elderly, and immunocompromised patients [1,2,3,4,5,6]. *K. pneumoniae* belongs to the ESKAPE group of pathogens (ESCAPE is an acronym for Enterococcus faecium, *Staphylococcus aureus*, *K. pneumoniae*, *Acinetobacter baumannii*, *Pseudomonas aeruginosa*, *Enterobacter* spp.) [2,7] and to the top priority list of “critical” pathogens of the WHO [8], having been indicated as a major target for the development of new prevention and therapeutic strategies.

The global emergence of multidrug-resistant (MDR) strains, especially extended-spectrum β-lactamase (ESBL)- or/and carbapenemase-producing *K. pneumoniae* (CPKP), has become an ultimate challenge for public health [6,9]. Treatment options against CPKP are sparse and usually limited to only last-line antibiotics, if at all [5,9]. Some of the new therapeutic approaches under development are based on concepts of active and passive immunization against major surface antigens of *K. pneumoniae*−the capsular polysaccharide (CPS, K-antigen) and lipopolysaccharide (LPS, endotoxin, O-antigen) [5,10]. Several vaccine strategies targeting the most prevalent K-antigens of *Klebsiella* have been developed, particularly against K1 and K2 characteristics of hypervirulent strains [11,12,13].

Contrary to highly variable K-antigens (more than 80 types) [1,14,15], LPS represents the less variable antigen and is an important virulence factor, triggering the Toll-like receptor 4-dependent immune response. It consists of lipid A, core oligosaccharide, and O-specific polysaccharide (O-PS, O-antigen), with the latter part determining the O-serotype. As only 11 O-serotypes have been identified for *K. pneumoniae* to date (O1, O2a, O2ac, O2afg, O2aeh (called also O9), O3, O4, O5, O7, O8, and O12) [16,17,18], with two sub-serotypes (O3a, O3b) [1,19,20,21], O-antigens have been suggested as potential targets for active or passive immunization for decades [10].

Promising bactericidal and neutralizing monoclonal antibodies targeting the most common *K. pneumoniae* O-serotypes (O1, O2, O3, and O5) have recently been developed [19,20,21,22,23]. However, for the success of K/O-antigen-based treatment strategies, comprehensive knowledge on serotype distribution, novel serotypes, their structural diversity, and genotype–phenotype relationships among clinical isolates are mandatory. At present, classical structural analysis based on carbohydrate chemistry is efficiently supported by molecular biology and bioinformatics tools for K- and O-antigen typing [1,24], including Kaptive Web [14]. These tools identify and analyze the specific K- and O-loci encoding individual CPS or O-PS structures, respectively. The O-antigen biosynthesis of *K. pneumoniae* depends on genes of the *rfb* locus located between the *cps* and *hisI* genes [16,21]. To date, nine O-antigen gene clusters have been defined (for serotypes O1/O2, O3/O5, O4, O8, O12, OL101, OL102, OL103, and OL104) [1]. A survey of genome sequences indicated that the *rfb* loci of 83% of *K. pneumoniae* isolates specify the O1, O2, or O3 serotypes [16].

**Figure 1:**
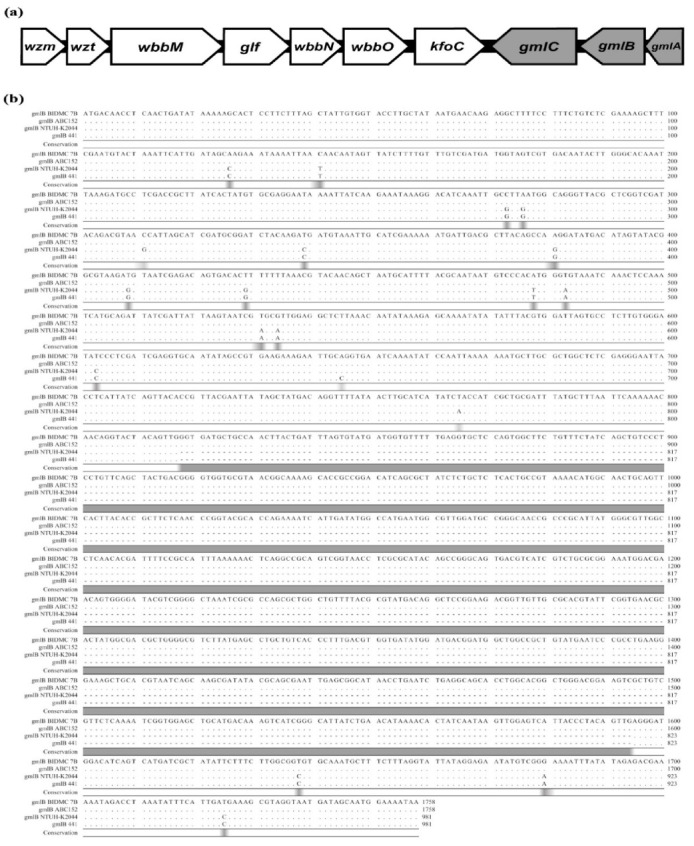
Organization and comparative analysis of *rfb* gene clusters: (**a**) Organization of the *rfb* cluster of *K. pneumoniae* O1/O2 variant 2. The *wzm* and *wzt* genes encode transmembrane and nucleotide-binding domains of an ABC transporter. The *wbbMNO* genes encode glycosyltransferases. The *glf* gene encodes UDP-galactopyranose mutase. The function of *kfoC* is unknown. The *gml* genes (highlighted in grey) encode the structural modification of D-galactan I; and (**b**) alignment of the *gmlB* genes of *K. pneumoniae* BIDMC 7B, ABC152, and two reference strains with *gmlABC* locus (NTUH-K2044 and 441). Grey areas mark regions of differences in nucleotide sequence. The alignment was performed using the CLC Main Workbench.

The O1/O2 *rfb* locus includes the following essential genes: (a) *wzm* and *wzt*, encoding transmembrane and nucleotide-binding domains of an ABC transporter; (b) *wbbMNO*, encoding glycosyltransferases; (c) *glf*, encoding the UDP-galactopyranose mutase which converts UDP-Gal*p* to UDP-Gal*f*; and (d) *kfoC*, which is of unknown function [1,16,21,24]. Moreover, the O1/O2 antigen gene cluster occurs in two variants: The O1/O2 variant 1 (v1; O2a, O2ac serotypes), characterized by the presence of D-galactan I, is encoded by the mandatory *wzm*-*wbbO* genes [1,21,25]. The second variant (v2; O2afg, O2aeh serotypes) carries an additional three genes, *gmlABC* (*gmlABD*, in the case of the O2aeh serotype) [16,20,21]. These genes are in opposite orientation and located downstream of *rfb* (Figure 1a). The *gmlABC* products are three putative glycosyltransferases, which modify D-galactan I {→3)-β-D-Gal*f*-(1→3)-α-D-Gal*p*-(1→} (O2a; O2v1) to branched polymers of {→3)-β-D-Gal*f*-(1→3)-[α-D-Gal*p*-(1→4)]-α-D-Gal*p*-(1→} (O2afg; O2v2) or {→3)-β-D-Gal*f*-(1→3)-[α-D-Gal*p*-(1→2)]-α-D-Gal*p*-(1→} (O2aeh) disaccharides, respectively [16,20,21]. O-PS of O1 contains D-galactan I and D-galactan II, built of {→3)-β-D-Gal*f*-(1→3)-α-D-Gal*p*-(1→} and {→3)-α-D-Gal*p*-(1→3)-β-D-Gal*p*-(1→} disaccharide repeating units, respectively. D-galactan II is encoded by a separate operon containing two genes located outside the *rfb* locus *wbbYZ* [1,21,24]. The O2 serotype is devoid of D-galactan II.

In this paper, we describe two clinical isolates of *K. pneumoniae* (strains BIDMC 7B and ABC152), in which Kaptive-based O-serotype prediction and O-antigen structural analysis reveal different O-serotypes. Molecular characterization was performed to explain the genotype–phenotype discrepancies as a result of insertion sequences (ISs) within their *rfb* regions. Further, large-scale in silico analysis of 8130 *K. pneumoniae* genomes available in public databases was performed, in order to assess the prevalence of such insertions in *rfb* of *K. pneumoniae* O1 and O2 genomes.

## 2. Results

### 2.1. O-Antigen Structures of the BIDMC 7B and ABC152 Strains Represent the O2 Variant 1 O-Serotype

**Figure 2:**
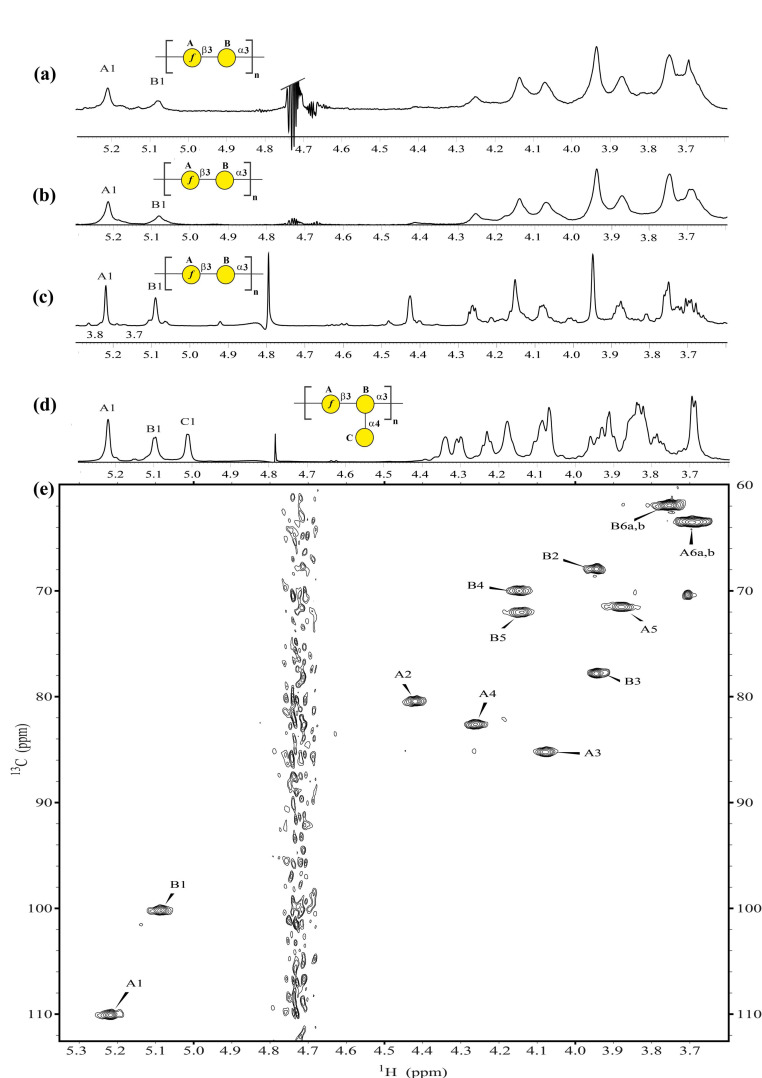
Comparative NMR analysis of *K. pneumoniae* O2v2 and O2v1 O-antigens. ^1^H HR-MAS NMR spectra of the lipopolysaccharides from (**a**) *K. pneumoniae* BIDMC 7B and (**b**) ABC152; and (**c**) the O-specific polysaccharides of *K. pneumoniae* Kp26 strain (O2v1) and (**d**) the PCM-27 strain (O2v2); (**e**) ^1^H,^13^C HSQC-DEPT NMR spectrum of the BIDMC 7B strain (O2v1). The capital letters refer to protons and/or carbons of O-PS carbohydrate residues, as shown in Table 1. The symbol Nomenclature for Graphical Representation of Glycans is used for O-PS visual representation: 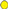 Galactose; 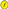 Galactofuranose to show O-specific polysaccharide repeating units: →3)-β-D-Gal*f*-(1→3)-α-D-Gal*p*-(1→ (D-galactan I, O2 variant 1) and →3)-β-D-Gal*f*-(1→3)-[α-D-Gal*p*-(1→4)]-α-D-Gal*p*-(1→ (O2v2) [26].

The O-antigen chemical structures of the *K. pneumoniae* BIDMC 7B and ABC152 clinical isolates were characterized by nuclear magnetic resonance (NMR) spectroscopy on the native isolated LPS. LPS was extracted from bacteria with yields of 0.32% and 0.70% for BIDMC 7B and ABC152, respectively, and then analyzed by the high-resolution magic angle spinning (HR-MAS) ^1^H, ^13^C NMR spectroscopy (Figure 2a,b,e).

Proton and carbon signals of the O-PS region of LPS prevailed in the NMR spectra. The complete assignments of ^1^H and ^13^C resonances for both O-PS (Table 1) were performed by interpretation of the NMR spectra, including comparison of the ^1^H,^13^C HSQC-DEPT spectrum (Figure 2e) with spectra for the reference O-PS structures of *K. pneumoniae* Kp26 (O2v1) and PCM-27 (O2v2) isolates (Figure 2c,d) [21].

The phenotype analysis demonstrated that the O-PS of the BIDMC 7B LPS had an O2v1 serotype structure, characterized by the [→3)-β-D-Gal*f*-(1→3)-α-D-Gal*p*-(1→] disaccharide as the non-modified D-galactan I repeating unit (Figure 2a,e; Table 1). The same D-galactan I structure was determined for the ABC152 LPS (Figure 2b; Table 1). The lack of a terminal α-D-Gal*p* residue, characteristic for the O2v2 serotype (Figure 2d, C1 signal), was confirmed for both isolates. Their NMR spectra were comparable to those recorded for the *K. pneumoniae* Kp26 O-PS (O2v1 serotype). The comparison of ^1^H, ^13^C HSQC-DEPT spectra of BIDMC 7B (Figure 2e) and ABC152 showed a complete overlay, confirming the identity of these O-PSs (Table 1).

### 2.2. Disruption of the gmlB Gene by IS Affects the O-Antigen Phenotype in BIDMC 7B and ABC152

Kaptive-based O-serotyping was performed with whole-genome sequences of both strains [14]. Contrary to the structural analysis, the O-serotypes were predicted to be O2v2 with high match confidence, according to the Kaptive measures of match quality. However, the BIDMC 7B and ABC152 *rfb* clusters demonstrated an increased size (by 777 bp) when compared to the reference sequences in the *Klebsiella* O-locus primary reference database in Kaptive. The *gmlABC* genes showed 100%, 90.79%, and 97.33% identity, respectively, to those in the Kaptive reference database.

Molecular analysis of BIDMC 7B and ABC152 was performed to explain the discrepancies observed between the O-antigen phenotype and the Kaptive-predicted O-serotype. The alignment of the *gmlB* genes from BIDMC 7B, ABC152, and from the O1/O2v2 reference strains *K. pneumoniae* NTUH-K2044 and 441 are shown in Figure 1b. This comparison shows the disruption of *gmlB*s in both strains by an identical IS element, IS*R1*, whereas other genes in the *rfb* locus were intact, in comparison to the reference strains. These results indicated that the IS*R1* disruption completely inactivated the GmlB glycosyltransferase gene, resulting in biosynthesis of the O2v1 instead of O2v2 structure, and thus, being the likely reason for the discrepancy between the actual O-antigen phenotype and Kaptive-based predictions.

### 2.3. ISs Occur in O2v2 and O1v2 K. pneumoniae Isolates—in Silico Study

In order to assess the occurrence of IS elements in *rfb* loci of *K. pneumoniae*, 8130 genome sequences available in the public domain were analyzed (Supplementary Material Table S1). Based on the Kaptive results of the O-serotyping (Supplementary Material Table S1, column B), 2281 isolates (≈ 28%) were predicted to be O2v2, and 839 isolates (≈ 10%) to be O1v2. For O2v2, 55 genomes (≈ 2.40%) revealed a significant difference in length of the *rfb* region (≥ 400 bp), of which 49 genomes were of sufficient quality for further analysis (Supplementary Material Table S2). The presence of different ISs (e.g., IS*R1*, IS*903B*, IS*Kpn14*, or IS*Kpn26*) were identified in several genes of these loci; namely, *gmlBC*, *kfoC*, *wbbMNO*, *glf*, *wzm*, and *wzt* (Table 2). In several isolates, the same or two different ISs interrupted two genes; namely, *gmlB* or *gmlC* and *wbbO*, or *wbbM* and *wbbO*.

Among the 839 Kaptive-identified O1v2 isolates, significant length discrepancies (≥700 bp) occurred in the *rfb* region of six isolates (≈0.7%), one of which was excluded due to the low quality of reads (Supplementary Material Table S3). Selected *rfb* genes of these isolates, namely *gmlABC*, *wbbM*, and *wzm*, were interrupted by IS*5*, IS*102*, IS*903B*, or IS*Kpn14* (Table 3). Two and one O1v2 isolates, two and four genes, respectively, were disrupted simultaneously. In both the O2v2 and O1v2 groups, the same ISs were observed at the same positions of the same genes in several isolates, suggesting their close genetic relatedness. The *gmlB*:IS*R1* (nt 818) disruption of the studied isolates BIDMC 7B and ABC152 was found in four other genomes.

**Figure 3:**
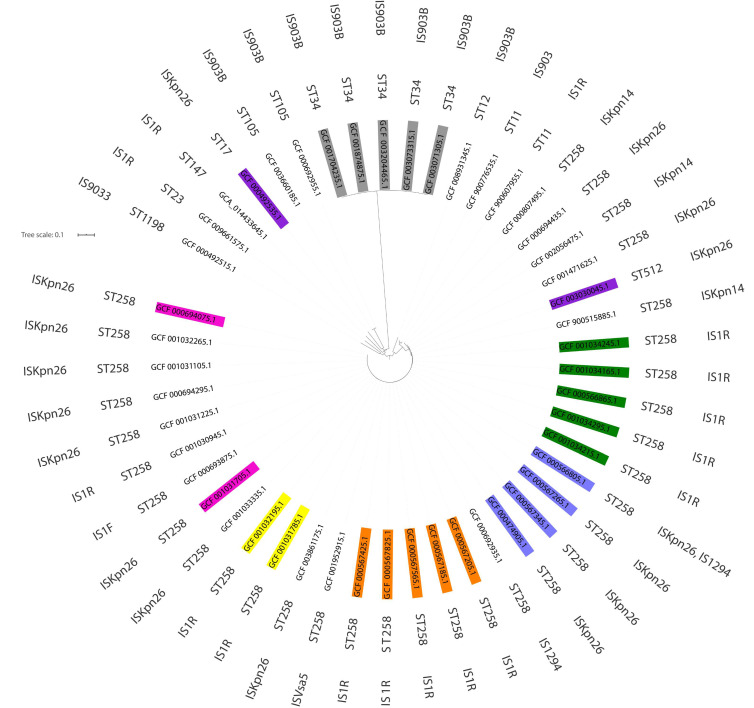
Results of clonality (MLST) and phylogenetic analyses of O2v2 *K. pneumoniae* genomes characterized by ST and insertion sequences distribution. Each color indicates closely related strains characterized by genetic similarity, shown in detail in Supplementary Material Table S2. Separately analyzed isolates are not colored.

In order to sort out the approximate number of independent IS insertions into the *rfb* loci of the available *K. pneumoniae* O2v2 and O2v1 genomes, clonality (MLST) and phylogenetic analyses were performed on the isolates using the ABC152 strain as a reference (Supplementary Material Tables S2 and S3; Figure 3). These confirmed that some individual disruptions within the *rfb* locus have spread in *K. pneumoniae* populations clonally with specific lineages, indicating single IS insertion events at their origins. This was demonstrated by clusters of O2v2 ST258 isolates with *kfoC*:IS*R1* (nt 656) or *wbbM*:IS*R1* (nt 1,881) disruptions, ST258 with double *gmlB*:IS*Kpn26* (nt 453) plus *wbbO*:IS*Kpn26* (nt 490) disruptions, or ST34 with *gmlC*:IS*903B* (nt 7,956). In some cases, an additional IS insertion likely marked on-going diversification within a lineage, such as *wbbO*:IS*903B* (nt 130) in ST34 with *gmlC*:IS*903B* (nt 7,956). An interesting case was the *gmlB*:IS*R1* (nt 818) disruption in the study isolates BIDMC 7B and ABC152, which was observed also in four others. BIDMC 7B plus the four others formed a closely related cluster of ST258 isolates. ABC152 was of a non-related ST147, suggesting a horizontal transfer and recombination event. A similar case was represented by the disruption *wbbO*:IS*Kpn26* (nt 1,014), present in two ST258 and ST512 close relatives, as well as a non-related ST17 isolate. Based on these results, it may be assumed that IS disruptions within the *rfb* loci in *K. pneumoniae* O2v2 and O2v1 genomes might have occurred at least ≈35 and ≈10 times, respectively (Table 2 and Table 3).

## 3. Discussion

Owing to their universality, reproducibility, varied resolution, and standardized high-throughput protocols, molecular biology methods have become an excellent tool for pathogen characterization, finding wide application in microbiology diagnostics and surveillance. In recent years, these have been revolutionized by WGS, an increasingly common approach used in public health laboratories for the control of antimicrobial resistance or bacterial genotyping. At present, WGS is also successfully used to complement laborious structural chemical analyses, such as those of microbial surface antigens, being key pathogenicity factors as well as critical targets for vaccines and therapeutic strategies [27,28,29].

As the molecular genetics of the *K. pneumoniae* O- and K-antigen biosynthesis has been well-elucidated, new O-genotyping techniques have been demonstrated to be useful for serotyping. There are several useful examples of tracking O- or K-antigen diversity among *K. pneumoniae* isolates [1,14,24]. For example, Fang et al. used a PCR-based O-genotyping approach to explore the distribution of the O-antigen genetic determinants in 87 clinical *K. pneumoniae* strains, showing a high prevalence of O1 (≈ 57%), followed by the O2a, O3, and O5 O-genotypes [24]. Follador et al. analyzed over 500 whole-genome sequences and reached a similar conclusion: that O1, O2, and O3 serotypes were the most common, with approximately 80% of all isolates [1]. Finally, Wick et al. presented the user-friendly Kaptive Web, an online tool for the rapid typing of *K. pneumoniae* surface polysaccharide loci, and demonstrated its utility using more than 500 *K. pneumoniae* genomes [14].

Kaptive Web-supported differentiation between *K. pneumoniae* O1/O2v1 and O1/O2v2 serotypes based on two steps: First, Kaptive recognizes the serotype by searching for the D-galactan-II-encoding genes (*wbbY*, *wbbZ*) characteristics of the O1 serotype. Second, the O1 and O2 serotypes are distinguished by the analysis of genes found in the *rfb* cluster. Finally, these are reported as variant v1 or v2 [14]. As the final result, the tool prediction is accompanied by length discrepancy information, which may indicate the possibility of some rearrangements in the *rfb* region.

In this study, we presented two cases of genotype–phenotype discrepancies for O-antigens in the *K. pneumoniae* clinical isolates BIDMC 7B and ABC152, the actual phenotype of which was O2v1, whereas Kaptive predicted O2v2. However, the tool provided an alert about “length disruption” within the *rfb* region and recommended further analyses. In the case of BIDMC 7B and ABC152 isolates, the structural analysis by the HR-MAS NMR spectroscopy proved the O-antigen structure to be O2v1 (Figure 2). The IS*R1* element was identified in the *gmlB* gene, one of the three responsible for the D-galactan I conversion from v1 to v2. The presence of IS, actual O-antigen structures, and the lack of other obvious differences between the analyzed genomes and O2v1 reference strains indicated that the IS disruption was the reason for the discrepancy between the O-antigen phenotype and the Kaptive-based prediction. The large-scale in silico analysis of publicly available genomes of *K. pneumoniae* O2v2 and O1v2 clearly showed that various insertions have occurred in several *rfb* fragments, possibly causing similar divergences between the O-serotype prediction and phenotype. A variety of ISs have been identified, including the common elements IS*R1*, IS*Kpn14*, IS*Kpn26*, and IS*903B*.

As only structural verification in each strain could provide definite proof of the O-phenotype, the in silico survey only suggested the influence of IS on the O-antigen chemical structure. By analogy with the BIDMC 7B and ABC152 strains, fourteen O2 strains (e.g., ASM170423, CHS57, and IS39) revealed ISs in the *gmlABC* region with higher prevalence of *gmlB* and *gmlC* disruptions, likely representing similar genotype–phenotype discrepancies. Three O2 strains with an IS in the *gmlABC* region had additional disruption within the *wzm*–*wbbO* region, suggesting failure of the O-antigen biosynthesis and the rough form of LPS, devoid of O-PS (i.e., ASM307130, UCI 38, and BIDMC 13). Other identified cases also suggested O-antigen biosynthesis failure, including O1v2 isolates (Table 2 and Table 3). Regarding the genetic background of O1 and O2 antigen biosynthesis, the presence of an IS in the O-locus may influence the O-antigen phenotype by: (i) O2v2 to O2v1 or O1v2 to O1v1 conversion; or (ii) conversion from smooth to rough LPS. It is noteworthy that the results obtained from Kaptive Web depended on the IS location. In the case of gene disruption or frameshift mutation, the results will indicate the lack of an enzyme specific for the analyzed serotype, which may contribute to the false serotype prediction by the algorithm. For instance, the Kaptive results for the isolate IS39 indicated the absence of the *wbbM* and *gmlABC* genes (Table S2). Detailed analysis of the *rfb* region has shown the presence of these genes with a *gmlC* IS disruption and point mutations in the other three genes. Although Kaptive suggests the possibility of the presence of IS by reporting differences in length discrepancy, it is worth analyzing the nucleotide sequence of the *rfb* region more precisely, in order to exclude falsely predicted serotypes based on errors occurring during O-genotyping.

Sequence analysis of isolates from the database confirmed the occurrence of many IS insertions in the *rfb* region; however, the frequency of such events is hard to evaluate. Although there are no previous data on IS disruptions within the O-locus, in general, these elements are common in *K. pneumoniae* genomes [30]. The hyperepidemic clone ST258, characterized by the notorious production of KPC-type carbapenemases and extensive drug resistance, has more ISs than an average *K. pneumoniae* isolate of another ST [30,31]. Several IS types are especially frequent in ST258, such as IS*Kpn26* [30,32]. In our study, the majority of O2 isolates identified belonged to ST258 (≈70%), and IS*Kpn26* was commonly found in these (≈50%). These data further emphasized the impact of ISs on the evolution of *K. pneumoniae* ST258; however, one must also consider the over-representation of ST258 genomes in public databases, resulting from the high clinical and epidemiological relevance of these organisms.

According to Adams et al., 94% of *K. pneumoniae* strains have at least one IS in their genome, where transposition of these elements within the genome causes rearrangements and may create new genotypes [30]. One consequence of an IS disruption of the *rfb* cluster genes may be the protection of bacteria against the host immune system. Although the presence of ISs in *gmlABC* genes may cause the phenotype–genotype discrepancy discussed above, the disruption of the genes determining D-galactan I elements may abolish the O-antigen synthesis, as in the case of the *wzm* or *wzt* genes, coding for ABC transporters [1,16,25]. IS elements in the *rfb* and/or *wbbYZ* operon can either inhibit the expression of the O-antigen on the surface of the bacterial cell [24] (resulting in the rough phenotype) or cause the switch from one serotype to another. In both cases, the change in phenotype can alter or impair the virulence of the bacteria [33]. Structural large-scale analysis of *K. pneumoniae* isolates could determine the consequences of ISs in the *rfb* region and their effects on bacterial antigenicity and host interactions. Such changes can significantly affect the ability of bacteria to survive during antibacterial therapies; for example, by changing the surface antigens and virulence in regard to reactivity with the complement, antibodies, or phage resistance [34]. The antigenic drift of LPS can be a way by which bacteria avoid the immune system. This is the case, for example, in the *Salmonella* species, the O-antigen composition of which affects the host–pathogen interactions during infection. Strains belonging to one serovar can have a different repertoire of O-antigen-modifying genes. Moreover, their expression is different depending on the phase variations. The *gtrABC* operon acquired by horizontal gene transfer is such a set of genes for modification of the *Salmonella* O-antigen. These genes encode proteins showing functional homology to the glycosyltransferases encoding by the *gmlABC* genes cluster in *K. pneumoniae* [35,36].

This study showed that some *K. pneumoniae* isolates, flagged by Kaptive Web as having length discrepancy within the *rfb* locus and advised for further analysis, may be basically mis-O-serotyped by the tool. As a methodology for the identification of an actual O-antigen phenotype is not broadly available, we assume that the development of the Kaptive algorithm in that direction would increase its high quality and usefulness, particularly for inexperienced users. Such development might be based on broader studies of isolates with non-clear O-serotyping results or identified O-genotype–phenotype discrepancies, with the use of structural analysis to precisely elucidate the O-genotype–phenotype relationships.

As O- and K-antigens represent target molecules for therapeutic strategies against *Klebsiella* infections, it is important to broaden our knowledge about genotype–phenotype relationships. Filling all detected gaps will improve serotype predictions based on bioinformatic tools. Exact fast prediction will enable the monitoring of *K. pneumoniae* antigen drift, which is vulnerable to selective pressure by therapies and vaccines.

## 4. Materials and Methods

### 4.1. Bacteria and Growth Conditions

*K. pneumoniae* BIDMC 7B (urine isolate) was obtained through BEI Resources, NIAID, NIH: “*Klebsiella pneumoniae*, strain BIDMC 7B, NR-41923”, as a reagent bought as a part of the Klebsicure-Eurostars project (no. E!7563). Strain ABC152 (urine isolate) was recovered from the Abu Dhabi Hospital (UAE) in 2013, kindly provided by Agnes Pal-Sonnevend and Tibor Pal from the United Arab Emirates University. Both strains were selected for a previous large-scale serotyping study (unpublished results), due to the inconsistency between the lack of LPS reactivity with O2v2-specific monoclonal antibody [21] and PCR results showing the presence of *gmlABC* genes. Bacteria were grown on Trypcase Soy Agar plates. For semi-preparative scale LPS preparation, the strains were cultured in Luria–Bertani (LB) broth in 500 mL flasks with shaking (at 37 °C), inactivated overnight with 3% formalin at 22 °C, then harvested by centrifugation, washed with water, and freeze-dried.

### 4.2. O-Specific Polysaccharides

*K. pneumoniae* PCM-27 and Kp26 O-specific polysaccharides were obtained from the Laboratory of Microbial Immunochemistry and Vaccines in the Ludwik Hirszfeld Institute of Immunology and Experimental Therapy, PAS (Wroclaw, Poland) and isolated as previously described [21].

### 4.3. LPS Preparation

LPS of *K. pneumoniae* strains BIDMC 7B and ABC152 were isolated by hot phenol/water extraction [37] and purified by dialysis and ultracentrifugation, as described elsewhere [38], followed by freeze-drying.

### 4.4. NMR Spectroscopy

All NMR spectra were obtained at 298 K using an Avance III 600 MHz (Bruker BioSpin GmbH, Rheinstetten, Germany) spectrometer equipped with a PH HR MAS probe (LPS analysis) or 5 mm QCI cryoprobe with z-gradients (O-PSs analysis). NMR spectra of isolated O-PSs were obtained in ^2^H_2_O, processed and analyzed as described previously [39]. For high-resolution magic angle spinning (HR-MAS) NMR spectroscopy, LPS (3–4 mg) was suspended in ^2^H_2_O and placed into the ZrO_2_ rotor. Acetone was used as an internal reference (δ_H_/δ_C_ 2.225/31.05 ppm) for both O-PS and LPS spectra [21]. The processed spectra (^1^H, ^13^C HSQC-DEPT, ^1^H, ^1^H COSY, and TOCSY) were assigned with the use of NMRFAM-SPARKY (v1.2, NMRFAM, Madison, Wisconsin, USA) [40].

### 4.5. DNA Isolation

Genomic DNA of the BIDMC 7B and ABC152 strains were extracted from overnight cultures with the Genomic Mini kit (A&A Biotechnology, Gdynia, Poland).

### 4.6. DNA Library Preparation and Sequencing

Libraries were prepared using the Nextera XT DNA Library Preparation Kit (Illumina Inc., San Diego, California, USA) and sequenced on an Illumina MiSeq platform (Illumina Inc., San Diego, CA, USA).

### 4.7. Sequence Analysis

The obtained reads were trimmed by Trimmomatic v0.39 [41]. The genomes of the BIDMC 7B and ABC152 strains were assembled using SPAdes v3.12 [42]. Assembled genomes of the BIDMC 7B and ABC152 isolates were analyzed by the Kaptive Web algorithm (https://github.com/katholt/Kaptive) for in silico O-serotyping [14]. Sequences of the *rfb* gene clusters were compared with those of the reference strains *K. pneumoniae* NTUH-K2044 (serotype O1v2) and *K. pneumoniae* 441 (O1/O2v2) (GenBank accession numbers, AB117611 and LT174602, respectively) using the CLC Main Workbench version 20 software (https://digitalinsights.qiagen.com; Qiagen, Hilden, Germany). All *K. pneumoniae* whole-genome sequences available from GenBank (*n* = 8130, as of 3 December 2019) were downloaded and screened using Kaptive for the O1v2- and O2v2-predicted serotype isolates, and for the *rfb* region length discrepancies among these (excess of ≥ 700 bp and ≥ 400 bp, respectively). Genomes of all such isolates were typed by 7-loci MLST with Multi-Locus Sequence Typing 2.0 (http://cge.cbs.dtu.dk/services/MLST) [43]. Identification of ISs was performed by ISfinder (http://www-is.biotoul.fr) [44]. Subsequently, all putative O2v2 genomes with *rfb* length discrepancies were aligned against the strain ABC152, inferring SNP-based phylogeny with Parsnp v.1.2. [45]. The phylogenetic tree was visualized with iTOL (https://itol.embl.de) [46].

### 4.8. Data Availability

The sequence of the BIDMC 7B strain is available in GenBank under accession number JCNG00000000.1. The sequence of the ABC152 strain was submitted to the GenBank database under accession number JACENF000000000.

## Figures and Tables

**Table 1:** ^1^H and ^13^C NMR chemical shifts of O-specific polysaccharides from *K. pneumoniae* BIDMC 7B and ABC152 LPS.

			Chemical Shift (ppm)					
Strain	Residue	Description	H1 C1	H2 C2	H3 C3	H4 C4	H5 C5	H6a,b C6
BIDMC 7B	A	→3)-β-D-Gal*ƒ*-(1→	5.22 110.1	4.42 80.5	4.07 85.2	4.26 82.6	3.88 71.5	3.69^nr^ 63.5
	B	→3)-α-D-Gal*f*-(1→	5.09 100.2	3.94 67.8	3.94 77.8	4.14 70.1	4.14 72.0	3.75^nr^ 61.9
ABC152	A	→3)-β-D-Gal*ƒ*-(1→	5.22 110.0	4.42 80.4	4.07 85.2	4.26 82.6	3.88 71.5	3.69^nr^ 63.5
	B	→3)-α-D-Gal*f*-(1→	5.09 100.1	3.94 67.9	3.94 77.8	4.14 70.0	4.14 72.0	3.75^nr^ 61.9

nr—not resolved.

**Table 2:** Sequence type and location of IS elements in the *rfb* clusters of *K. pneumoniae* isolates selected on the basis of Kaptive-based O2v2 predictions ^a^.

Isolate	Assembly Accession Number	Sequence Type	Insertion Sequence	Gene	Position of the IS Element (From the First Nucleotide of CDS)
27097_7_178-2	GCF_900776535.1_27097_7_178-2_genomic	ST11	IS*903*	*wbbO*	228 bp
kpneu028	GCF_900607955.1_kpneu028_genomic	ST11	IS*R1*	*wbbM*	47 bp
ASM893134	GCF_008931345.1_ASM893134v1_genomic	ST12	IS*903B*	*kfoC*	267 bp
UCI 7	GCF_000492535.1_Kleb_pneu_UCI_7_V1_genomic	ST17	IS*Kpn26*	*wbbO*	1014 bp
ASM966157	GCF_009661575.1_ASM966157v1_genomic	ST23	IS*R1*	*wbbM*	1420 bp
ASM170423	GCF_001704235.1_ASM170423v1_genomic	ST34	IS*903B*	*gmlC*	7956 bp in *rfb* (CDS *gmlC* from 7996 bp)
ASM307130	GCF_003071305.1_ASM307130v1_genomic	ST34	IS*903B*	*wbbO*	130 bp
			IS*903B*	*gmlC*	7956 bp in *rfb* (CDS *gmlC* from 7996 bp)
ASM366018	GCF_003660185.1_ASM366018v1_genomic	ST105	IS*903B*	*wbbO*	182 bp
BIDMC 55	GCF_000692955.1_Kleb_pneu_BIDMC_55_V1_genomic	ST105	IS*903B*	*glf*	105 bp
ABC152	GCA_014433645.1	ST147	IS*R1*	*gmlB*	818 bp
BIDMC 7B	GCF_000567425.1_Kleb_pneu_BIDMC_7B_V2_genomic	ST258	IS*R1*	*gmlB*	818 bp
UCI 33	GCF_000566865.1_Kleb_pneu_UCI_33_V1_genomic	ST258	IS*R1*	*kfoC*	656 bp
CHS 139	GCF_001031785.1_Kleb_pneu_CHS139_V1_genomic	ST258	IS*R1*	*wbbM*	1881 bp
CHS 91	GCF_001030945.1_Kleb_pneu_CHS91_V1_genomic	ST258	IS*R1*	*wbbO*	165 bp
CHS 57	GCF_000694075.1_Kleb_pneu_CHS_57_V1_genomic	ST258	IS*Kpn26*	*gmlB*	4 bp
UCI 38	GCF_000566805.1_Kleb_pneu_UCI_38_V1_genomic	ST258	IS*Kpn26*	*gmlB*	453 bp
			IS*1294*	*wbbO*	900 bp
BIDMC 13	GCF_000567345.1_Kleb_pneu_BIDMC_13_V1_genomic	ST258	IS*Kpn26*	*gmlB*	453 bp
			IS*Kpn26*	*wbbO*	490 bp
CHS 71	GCF_000694295.1_Kleb_pneu_CHS_71_V1_genomic	ST258	IS*Kpn26*	*wbbO*	1080 bp
CHS 235	GCF_001033335.1_Kleb_pneu_CHS235_V1_genomic	ST258	IS*Kpn26*	*wbbO*	641 bp
ASM147162	GCF_001471625.1_ASM147162v2_genomic	ST258	IS*Kpn26*	*wbbO*	1014 bp
			IS*Kpn26*	*wbbM*	96 bp
CHS 105	GCF_001031225.1_Kleb_pneu_CHS105_V1_genomic	ST258	IS*Kpn26*	*wbbM*	1548 bp
ASM386117	GCF_003861175.1_ASM386117v1_genomic	ST258	IS*Kpn26*	*wbbM*	45 bp
			IS*Kpn26*		2156 bp
CHS 165	GCF_001032265.1_Kleb_pneu_CHS165_V1_genomic	ST258	IS*Kpn26*	*kfoC*	158 bp
MGH 51	GCF_000694435.1_Kleb_pneu_MGH_51_V1_genomic	ST258	IS*Kpn26*	*wzm*	315 bp
CHS 99	GCF_001031105.1_Kleb_pneu_CHS99_V1_genomic	ST258	IS*Kpn26*	*wzt*	470 bp
ASM205647	GCF_002056475.1_ASM205647v1_genomic	ST258	IS*Kpn14*	*kfoC*	782 bp
ASM80749	GCF_000807495.1_ASM80749v2_genomic	ST258	IS*Kpn14*	*kfoC*	440 bp
18174_7_5	GCF_900515885.1_18174_7_5_genomic	ST258	IS*Kpn14*	*wbbO*	377 bp
BIDMC 54	GCF_000692935.1_Kleb_pneu_BIDMC_54_V1_genomic	ST258	IS*1294*	*kfoC*	1186 bp
CHS 46	GCF_000693875.1_Kleb_pneu_CHS_46_V1_genomic	ST258	IS*F1*	*wbbM*	1138 bp
ASM195291	GCF_001952915.1_ASM195291v1_genomic	ST258	IS*Vsa5*	*wbbN*	133 bp
ASM303004	GCF_003030045.1_ASM303004v1_genomic	ST512	IS*Kpn26*	*wbbO*	1014 bp
UCI 8	GCF_000492515.1_Kleb_pneu_UCI_8_V1_genomic	ST1198	IS*9033*	*wbbM*	1667 bp
IS39	GCF_000529425.1_IS39v1_genomic	unknown	IS*102*	*gmlC*	7933 bp in *rfb* (CDS *gmlC* from 7996 bp)

^a^: O-serotype predictions were performed using the Kaptive Web tool [14].

**Table 3:** Sequence type and location of IS elements in the *rfb* clusters of *K. pneumoniae* isolates selected on the basis of Kaptive-based O1v2 predictions ^a^.

Isolate	Assembly Accession Number	Sequence Type	Insertion Sequence	Gene	Position of the IS Element (From the First Nucleotide of CDS)
ASM492431	GCF_004924315.1_ASM492431v1_genomic	ST23	IS*102*	*gmlC*	1217 bp
ASM275277	GCF_002752775.1_ASM275277v1_genomic	ST29	IS*102*	*gmlB*	472 bp
			IS*903B*	*gmlC*	526 bp
ASM296687	GCF_002966875.1_ASM296687v1_genomic	ST34	IS*5*	*wzm*	315 bp
			IS*903B*	*wbbM*	465 bp
			IS*903B*	*gmlB*	926 bp
			IS*903B*	*gmlC*	143 bp
ASM290977	GCF_002909775.1_ASM290977v2_genomic	ST231	IS*Kpn14*	*gmlA*	95 bp

^a^: O-serotype predictions were performed using the Kaptive Web tool [14].

## Supplementary Materials

Supplementary materials can be found at https://www.mdpi.com/1422-0067/21/18/6572/s1. Table S1: Kaptive Web analysis results for the 8130 assemblies of *K. pneumoniae* isolates. Isolates belonging to the O2v2 serotype are marked in green. Isolates belonging to the O1v2 serotype are marked in blue; Table S2: Kaptive analysis results extracted for the 55 *K. pneumoniae* O2 variant 2 genomes characterized by IS occurrence. Each color indicates closely related strains characterized by genetic similarity. Separately analyzed isolates are not colored; Table S3: Kaptive analysis results extracted for the 5 *K. pneumoniae* O1 variant 2 genomes characterized by IS occurrence. Isolates belonging to the same strain are marked in blue.

## Abbreviations

MDR	Multidrug-Resistant
ESBL	Extended-Spectrum Beta-Lactamases
COSY	Correlation Spectroscopy
CPKP	Carbapenemase-Producing *K. pneumoniae*
CPS	Capsular Polysaccharide; K antigen
DEPT	Distortionless Enhancement by Polarization Transfer
HR-MAS	High Resolution-Magic Angle Spinning
HSQC	Heteronuclear Single Quantum Correlation
IS	Insertion Sequence
LPS	Lipopolysaccharide
MLST	Multi-locus Sequence Typing
NMR	Nuclear Magnetic Resonance
O-PS	O-specific Polysaccharide
PCR	Polymerase Chain Reaction
ST	Sequence Type
TOCSY	Total Correlation Spectroscopy
WGS	Whole-genome Sequencing
